# Sandwich EVAR occludes Celiac and Superior Mesenteric Artery for Infected Suprarenal Abdominal Aortic Aneurysm Treatment

**DOI:** 10.1155/2018/4037683

**Published:** 2018-05-10

**Authors:** Supatcha Prasertcharoensuk, Narongchai Wongkonkitsin, Parichat Tunmit, Su-a-pa Theeragul, Anucha Ahooja

**Affiliations:** ^1^Department of Surgery, Khon Kaen University, Khon Kaen, Thailand; ^2^Department of Radiology, Khon Kaen University, Khon Kaen, Thailand

## Abstract

*Introduction*. Infected aortoiliac aneurysms are rare, representing only 1% to 2% of all aortic aneurysms; we present a case of infected suprarenal aortic aneurysm with a nearly occluded celiac artery and superior mesenteric artery treated using an endovascular technique to preserve collateral in the retroperitoneal space from the inferior mesenteric artery for supplying visceral organs.

## 1. Introduction

Infected aortoiliac aneurysms are rare, representing only 1% to 2% of all aortic aneurysms [1]. One study in an Asian population found that infected abdominal aortic aneurysms accounted for 13.6% of cases and were associated with morbidity and mortality rates of 21%–44% [2]. Infected abdominal aortic aneurysm may present in various signs and symptoms that are not specific to infected abdominal aortic aneurysm, which may lead to misdiagnosis and delay treatment [3]. Treatment options for infected suprarenal abdominal aortic aneurysm include medication, open in situ graft repair, and hybrid endovascular repair [4].

There are also several surgical procedures used to treat the condition, including surgical debridement with in situ graft interposition and omental wrapping, aneurysm exclusion and extra-anatomic (axillofemoral) bypass, and aneurysmectomy with polytetrafluoroethylene (PTFE) graft interposition. The main factors associated with outcomes are whether or not appropriate surgical intervention was performed, whether or not the patient was treated with proper antibiotics, and whether or not the IAAA was ruptured. Here, we present a case of infected suprarenal aortic aneurysm with a nearly occluded celiac artery and superior mesenteric artery treated using an endovascular technique to preserve collateral in the retroperitoneal space from the inferior mesenteric artery for supplying visceral organs. We also report on the clinical outcomes of this technique. To our knowledge, this is the first report to describe a total endovascular procedure treatment of the infected suprarenal aortic aneurysm using the sandwich technique without preserved flow to both celiac artery and superior mesenteric artery.

## 2. Case Report

**Figure 1:**
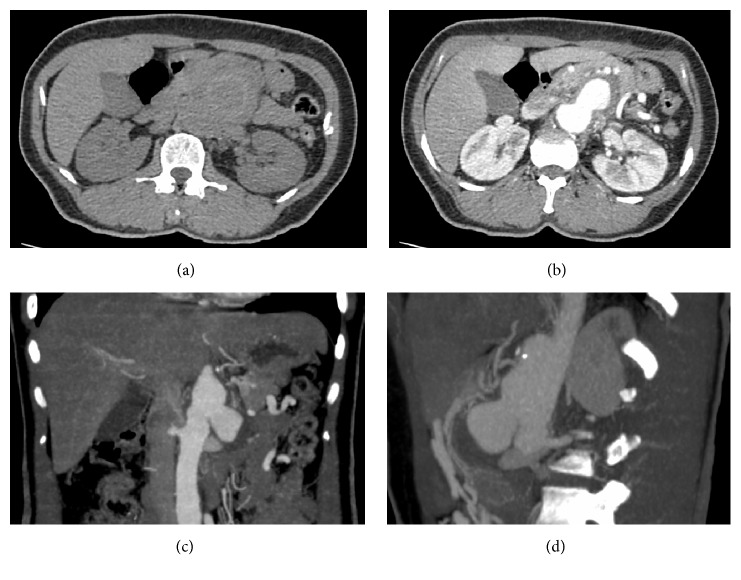
Crescent sign of suprarenal aortic aneurysm (a). A-phase shows saccular aneurysm behind the pancreas (b). Coronal view: suprarenal saccular aneurysm mainly on the left side (c). Sagittal view: aneurysm bulge anteriorly displacing the pancreas (d).

A 33-year-old woman presented with abdominal pain and having had a low-grade fever for three days. Computed tomographic angiography revealed a saccular aneurysm at the paravisceral aortic region, a nearly occluded celiac artery, and superior mesenteric artery with a large patent inferior mesenteric artery (7.3 mm), which provided a large collateral in the retroperitoneal space for supplying visceral organs, as shown in Figure 1. She had no history of trauma, pancreatitis, diabetes mellitus, recent medical or dental procedures, or drug abuse.

On examination, her blood pressure was 104/64 mmHg and she was febrile. The central abdomen was tender with a four-centimeter palpable pulsatile mass. The patient's pulse at all of the lower limbs was normal. Investigations revealed haemoglobin levels of 12.4 g/dL and a white cell count of 11.9 × 10^3^/uL with neutrophilic change (77.8%) and without band form. Her platelet count was 317 × 10^3^/ml, erythrocyte sedimentation rate (ESR) was 90 mm/hr, and C-reactive protein level was 8.9 mg/l. Blood and urine cultures showed no growth. Results of transthoracic echocardiography were normal.

**Figure 2:**
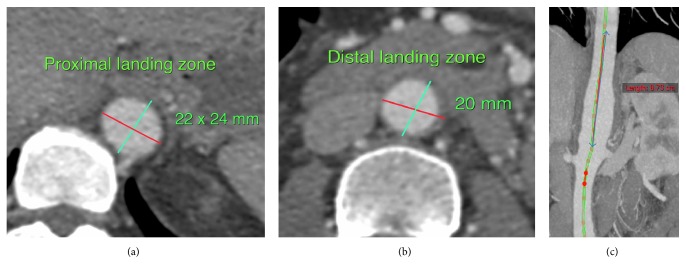
Proximal landing zone with diameter of 22 mm (a). Distal landing zone with diameter of 20 mm (b). Coronal view shows inflammatory and saccular aneurysm part (c).

We diagnosed this patient as having an infected suprarenal aortic aneurysm, due to CTA findings showing saccular aneurysm and periaortic inflammation in the visceral segment of the aorta, even though her hemoculture was negative. As such, intravenous ceftazidime and clindamycin were empirical to cover* Burkholderia pseudomallei* and* Salmonella species* which are endemic infections in the Northeast of Thailand. The patient experienced ongoing pain and developed intermittent pyrexia of up to 38.4°C. Blood cultures remained negative and her white cell count reached 14.5 × 10^3^/uL with neutrophilic change (89.7%) and without band form on the fourth day after admission. Measurement of the aorta found the proximal landing zone to be at descending aorta level T10-11 with a diameter of 24 mm and the distal landing zone to be 20 mm in diameter above the IMA. The distance from the proximal landing zone to just above renal arteries was 90 mm, as shown in Figure 2.

**Figure 3:**
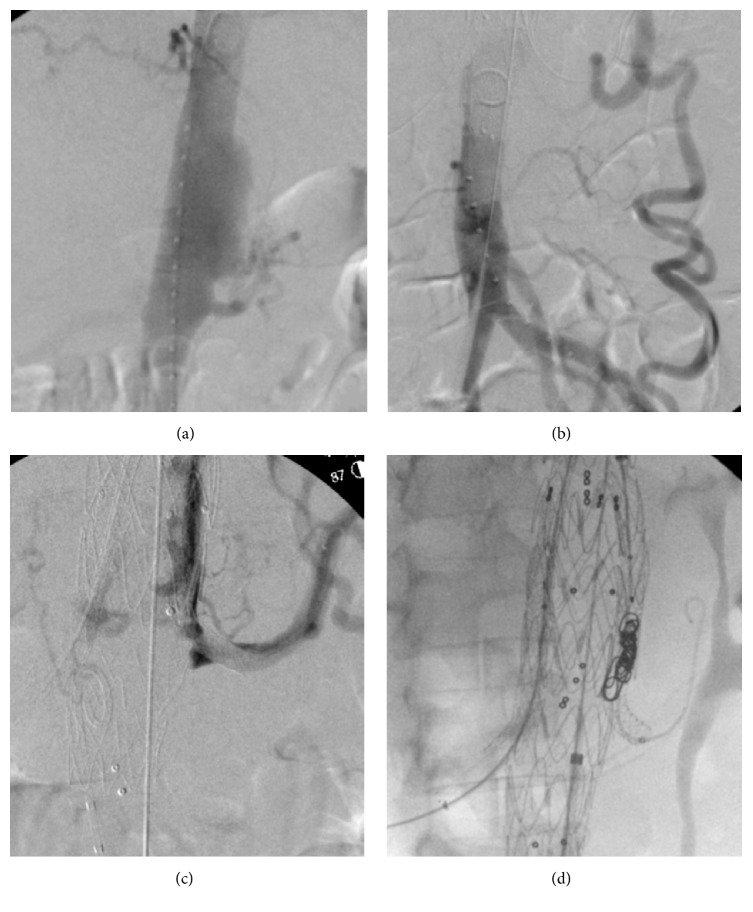
Intraoperative angiogram shows suprarenal saccular aneurysm with nearly occluded celiac and superior mesenteric artery (a). Large patent of inferior mesenteric artery gives blood supply to whole abdomen (b). Endoleak type III from left gutter (c). Extended gutter and coil embolization between left renal stent and aortic stent (d).

**Figure 4:**
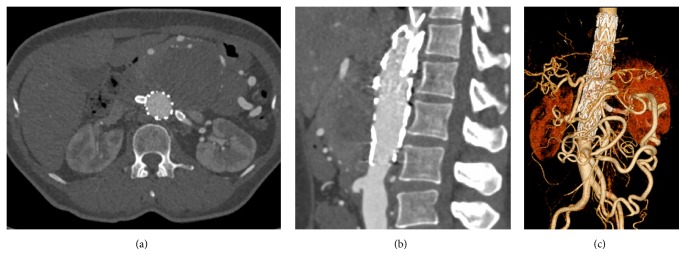
CTA: axial view, no endoleak (a). Sagittal view: stent placed superior to inferior mesenteric artery orifice (b). Reconstruction view: adequate flow from the inferior mesenteric artery to supply the celiac artery and superior mesenteric artery.

**Figure 5:**
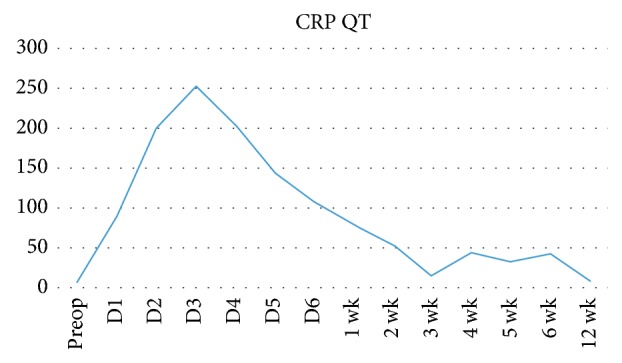
C-reactive protein levels from preoperation to 12 weeks after endovascular aneurysm repair (EVAR) by using the sandwich technique.

The patient underwent EVAR using a sandwich technique; the first aortic stent graft (VAMC2828C100TE) was deployed above left renal artery followed by advance guidewire to select both renal arteries in order to place the balloon covered stents (Begraft 6 × 58) to both sides. Then another endovascular aortic stent (ETTF2828C70EE) was simultaneously deployed to sandwich the renal artery stents. First, in complete aortogram, there was evidence of endoleak type 3 between left renal artery stent and endovascular aortic stent. We extended both renal stents and inner endovascular aortic stent (VAMC2828C100TE) to extend gutter between stents up to 50 mm and coil gutter. In final aortogram, there is only endoleak type 2, as shown in Figure 3. Postoperative management consisted of the prescription of six weeks of intravenous antibiotic (ceftazidime plus clindamycin) and ciprofloxacin and clindamycin orally for the rest of the patient's life. At follow-up after one month and six months, the patient had no abdominal pain and had gained a significant amount of weight. Computed tomographic angiography results revealed that the aneurysm had shrunk, as shown in Figure 4 (three months after surgery). We also found that C-reactive protein (a biomarker) levels were dramatically lower after the aneurysm was repaired and returned to normal after three weeks (Figure 5). At the one-year follow-up, the patient was asymptomatic and had no bowel ischemia.

## 3. Discussion

Infected abdominal aortic aneurysms account for 2% of abdominal aortic aneurysms. Infected abdominal aortic aneurysms at the suprarenal abdominal aorta are extremely rare according to case reports, reports on small cases series, and literature review [5–8]. The common causative organism is* Salmonella* spp. [9, 10]. Treatment outcomes of IAAA may be associated with an aggressive debridement with in situ prosthetic reconstruction accompanied by prolonged antibiotic therapy and often lifelong suppressive oral antibiotics. However, according to a review based on several case reports, aneurysmectomy with aggressive debridement is reported as having a survival rate that is falsely high [11]. However, this operation is time-consuming, requires a significant number of technically demanding anastomoses to visceral vessels, and is usually associated with bypass-related complications such as renal failure, intestinal ischemia, and paraplegia. Hybrid endovascular repair, combining the open renovisceral debranching and endovascular stenting, is reserved for patients who are in high risk when standard open procedures are approached [12]. In this case, the patient in this study had a large number of collaterals in retroperitoneum, meaning that the debranching procedure may have cut off valuable collaterals that make visceral ischemia, so we abandoned the use of this procedure.

Endovascular repair of suprarenal infected abdominal aortic aneurysm is even more complex because concomitant renovisceral debranching is usually required. There are few clinical cases of total endovascular repair of suprarenal infected abdominal aortic aneurysm. A total of six cases have been reported in the English literature [13–15]. The largest case series by Sörelius et al. in 2009 reported on three cases of endovascular repair of infected suprarenal abdominal aortic aneurysm. Previous studies have reported a mortality rate of 33% (3/9) with two cases of intestinal ischemia (2/9) and one case of sepsis (1/9) [16].

## 4. Conclusion

Sandwich EVAR can be used safely in cases of complex anatomy, in which emergency intervention is required.
